# Emotion Regulation, Fear of Hypoglycemia, and Diabetes Distress in Parents of Children with Type 1 Diabetes

**DOI:** 10.3390/bs16060942

**Published:** 2026-06-08

**Authors:** Anabela Vieira, Vasco Costa, Tânia Brandão

**Affiliations:** 1 School of Psychology, Ispa—Instituto Universitário, 1149-041 Lisbon, Portugal; anabelagordalinavieira@gmail.com; 2 William James Center for Research, Ispa—Instituto Universitário, 1149-041 Lisbon, Portugal; vcosta@ispa.pt

**Keywords:** type 1 diabetes, fear of hypoglycemia, parental diabetes distress, emotional regulation

## Abstract

Parents of children and adolescents with type 1 diabetes (T1D) are responsible for intensive daily disease management and often experience high levels of emotional distress. This study examined whether fear of hypoglycemia mediates the association between parents’ emotion regulation strategies and diabetes-related distress. Participants were recruited through Facebook and WhatsApp groups for parents of children and adolescents with T1D, and data was collected via self-report online questionnaires. A total of 102 parents, 92.2% mothers (aged 32–58 years) of children with T1D aged 8–17 years, completed measures of fear of hypoglycemia (Hypoglycemia Fear Survey—Parent Version), diabetes distress (Problem Areas in Diabetes-Parent Revised) and emotion regulation strategies (Emotion Regulation Questionnaire), along with a sociodemographic questionnaire. Four mediation models were tested using PROCESS, including cognitive reappraisal and expressive suppression as predictors and the worry and behavior subscales of fear of hypoglycemia as mediators. Results revealed a significant indirect effect of worry on the relationship between cognitive reappraisal and diabetes distress (*indirect effect* = −0.15, 95% CI [−0.35, −0.02]), highlighting worry as a potential mediator between these variables, while the direct effect was negative but non-significant. No significant indirect effects were found for expressive suppression on the behavior subscale (*indirect effect* = 0.12; 95% IC [−0.07; 0.36]) or on the worry subscale (*indirect effect* = 0.07; 95% IC [−0.08; 0.24]). These findings suggest that cognitive reappraisal may be associated with lower diabetes-related distress through lower levels of excessive worry about hypoglycemia. Clinically, the results highlight fear-related cognition can be a relevant intervention target, alongside emotion regulation skills, in psychosocial support programs for parents of youth with T1D.

## 1. Introduction

Type 1 diabetes (T1D) is an autoimmune disease characterized by the progressive destruction of pancreatic beta cells responsible for insulin production, which is essential for glucose uptake by body cells and for maintaining energy metabolism (Arroyo-Jousse et al., 2015). T1D is most commonly diagnosed in children, adolescents, and young adults (American Diabetes Association, 2024). Globally, approximately 589 million individuals are living with diabetes (International Diabetes Federation, 2025), and in Portugal, about 900,000 individuals had been diagnosed by December 2023 (Do Vale et al., 2024).

This chronic condition requires intensive and continuous daily management by parents, particularly when children are young. Key responsibilities include frequent blood glucose monitoring, administration of exogenous insulin, regulation of carbohydrate intake, and adjustment of physical activity, with increased risk of acute and long-term complications when glycemic control is suboptimal (Azimi et al., 2024; Sociedade Portuguesa de Diabetologia, 2023). These ongoing demands, combined with disruptions to family routines and limited access to formal and informal support, can contribute to elevated levels of psychological and emotional distress among parents (Mohan et al., 2024; Tong et al., 2022).

Parental diabetes distress (DD) is a condition-specific form of distress defined as a subjective emotional response to the burden imposed by the intensive and demanding daily management of a child’s diabetes (Arabiat et al., 2020). Although DD is not considered a psychiatric disorder (Fisher et al., 2014), it may present with symptoms that overlap with depression, anxiety, burnout, and other psychological difficulties, which often co-occur (Fisher et al., 2019; Kiriella et al., 2021). These symptoms may include sadness, fear, mood fluctuations, frustration, appetite and sleep disturbances, anxiety, social withdrawal, fatigue, interpersonal conflict, and feelings of loneliness (Pallayova & Taheri, 2014).

Studies indicate a high prevalence of DD among parents caring for children and adolescents with T1D, ranging from approximately 61% to 67%, while prevalence among youth ranges from 40% to 63% (Kahhan et al., 2025; Stapleton et al., 2022), with negative consequences for psychological well-being and quality of life in both parents and children (Noser et al., 2019). Therefore, identifying and promoting strategies to reduce parental DD is particularly important, as lower levels of DD may facilitate more adaptive emotional functioning and coping with diabetes-related demands (Carreras et al., 2019).

Emotion regulation (ER) is a dynamic process through which individuals influence which emotions they experience, when they experience them, and how these emotions are expressed (Gross, 1998). ER enables individuals to monitor and modulate emotional responses, supporting internal balance and coordination between emotional and cognitive processes (Cicchetti et al., 1995; Yang et al., 2024). Through regulation, individuals may modify emotional responses, enhance or reduce positive or negative affect, or alter behavioral reactions to emotional stimuli (Gross & Thompson, 2007).

In the present study, two ER strategies were examined: cognitive reappraisal and expressive suppression, both derived from Gross’s (1998) Process Model of Emotion Regulation. According to this model, ER strategies may be implemented at different points in the emotion-generative process (Gross, 2014; Gross & John, 2003). Antecedent-focused strategies are applied before the emotional response is fully activated; cognitive reappraisal is a central example of this class of strategies. Reappraisal involves modifying one’s interpretation of a situation to change its emotional impact (Gross, 2001, 2014). In contrast, response-focused strategies are applied after emotional response tendencies have already been generated; expressive suppression is a prototypical example of this type of strategy. Suppression refers to efforts to inhibit the outward expression of emotion through behavioral control (Gross, 2001; Gross & John, 2003; John & Gross, 2004).

Gross’s model is particularly relevant in pediatric T1D context, as parents are regularly exposed to emotionally demanding situations requiring constant vigilance and rapid decision making. Daily diabetes management often involves uncertainty regarding blood glucose fluctuations, concerns about hypoglycemic events, and responsibility for treatment-related decisions, all of which may activate intense emotional responses. In this context, antecedent-focused strategies such as cognitive reappraisal may help parents reinterpret stressful diabetes-related situations more adaptively, whereas response-focused strategies such as expressive suppression may involve inhibiting emotional expression without reducing internal distress, thereby influencing psychological adaptation to diabetes-related demands.

Beyond its general associations with lower levels of depression and anxiety in parents, particularly when cognitive reappraisal is used (Zimmer-Gembeck et al., 2022), effective ER may also contribute to reducing fear of hypoglycemia (Zhang et al., 2022), a central concern in the context of pediatric T1D management.

Hypoglycemia is one of the most acute and frequent complications of T1D in children and adolescents, and severe episodes may involve seizures, loss of consciousness, coma, or, in rare cases, death (Driscoll et al., 2016). Fear of hypoglycemia (FoH) is a condition-specific fear arising from parents’ concern related to two distinct dimensions: worry and behavior. The worry dimension reflects cognitive and emotional concerns, such as the fear and anxiety experienced by parents regarding the negative impact of hypoglycemia and their concern about the possibility of their child experiencing a hypoglycemic episode and their consequences. The behavior dimension assesses parents’ behaviors related to the prevention of hypoglycemic episodes and avoidance of hypoglycemic risk (Clarke et al., 1998; Gonder-Frederick et al., 2013). Higher levels of FoH have been associated with previous experiences of severe hypoglycemia (Patton et al., 2007), increased perceived responsibility for the child’s health (Streisand et al., 2005), and higher levels of parental DD (Monzon et al., 2021). A moderate level of FoH may be adaptive, as it can promote preventive behaviors such as more frequent blood glucose monitoring. However, FoH may also become maladaptive when parents adopt compensatory behaviors aimed at avoiding hypoglycemia that inadvertently increase the risk of hyperglycemia (Clarke et al., 1998). For example, to prevent hypoglycemic episodes, parents may increase their child’s carbohydrate intake, avoid intense physical activity, or reduce insulin administration (Böhme et al., 2013; Brod et al., 2012).

Prior research indicates significant associations between DD and the use of ER strategies (Bassi et al., 2023; Coccaro et al., 2021; Jaser et al., 2014). Higher levels of FoH problems have also been associated with greater DD and lower perceived parental self-efficacy in daily T1D management (Patton et al., 2011; Patton et al., 2019). Although some of these studies were conducted in adults with Type 1 and Type 2 diabetes, they remain relevant as a useful preliminary framework for understanding psychological processes involved in parental adaptation, particularly given the limited research focused specifically on parents of youth with T1D. Moreover, parents have a distinct caregiving role involving shared responsibility for diabetes management, increased vigilance, and ongoing concerns about their child’s safety and long-term health outcomes. In addition, interventions targeting ER processes, including mindfulness-based approaches, psychoeducation, communication and experience sharing, peer support, and support from specialized professionals, have demonstrated effectiveness in reducing FoH problems among parents (Aalders et al., 2018; Dehghankar et al., 2021; Marker et al., 2020).

Based on this evidence, the general objective of this study was to examine whether parents’ FoH mediated the association between ER strategies and DD in parents of children and adolescents with T1D. The specific objectives were to (a) assess the associations between cognitive reappraisal, expressive suppression, and DD, and (b) test the mediating role of FoH in these associations. Two hypotheses were formulated:

**H1.**  *Greater use of cognitive reappraisal would be associated with lower parental diabetes distress.*

**H2.**  *Greater use of expressive suppression would be associated with higher parental diabetes distress.*

**H3.**  *Fear of hypoglycemia dimensions (worry and hypoglycemia-related behaviors) will mediate the association between cognitive reappraisal and diabetes distress, such that greater use of cognitive reappraisal will be associated with lower FoH and, in turn, lower diabetes distress.*

**H4.**  *Fear of hypoglycemia dimensions will mediate the association between expressive suppression and diabetes distress, such that greater use of expressive suppression will be associated with higher FoH and, in turn, higher diabetes distress.*

## 2. Materials and Methods

### 2.1. Study Design

This study used a quantitative, cross-sectional, and correlational design to examine associations between emotion regulation strategies, FoH, and DD in parents.

### 2.2. Data Source and Participants

An a priori power analysis was conducted using G*Power (version 3.1.9.3, Düsseldorf, Germany), for a linear multiple regression model. Assuming a medium effect size (f^2^ = 0.15), an alpha level of 0.05, power of 0.95, and four predictors, the required total sample size was estimated at N = 89. The achieved sample size in the present study (N = 102) exceeded this requirement.

Participants were recruited through the dissemination of the study invitation in Facebook and WhatsApp groups for parents of children and adolescents with T1D. Data was collected between April and May 2024 using an online survey administered via Google Forms.2.5. It was a convenience sampling, with deliberate selection based on predefined eligibility criteria to ensure sample appropriateness for the study aims. Inclusion criteria were: (a) being a parent of a child or adolescent aged 1 to 17 years diagnosed with T1D, (b) residing in Portugal, and (c) having sufficient proficiency in Portuguese to complete the questionnaires. Exclusion criteria were: (a) the child or adolescent having a comorbid severe medical condition (assessed by self-report), and (b) the parent having a diagnosis of a severe psychiatric disorder (assessed by self-report).

The final sample comprised 102 participants, of whom 92.2% were mothers (n = 94) and 7.8% were fathers (n = 8), with ages ranging from 32 to 58 years (M = 44.58, SD = 5.01). Most participants were married or living with a partner (81.4%, n = 83), had completed undergraduate education (40.2%, n = 41), and reported middle socioeconomic status (82.4%, n = 84).

Children and adolescents with T1D were aged between 8 and 17 years (M = 12.67, SD = 2.58), with 48% female (n = 49) and 52% male (n = 53). Most were attending lower secondary education (3rd cycle; 39.2%, n = 40). The mean duration since T1D diagnosis was 5.92 years (SD = 3.52); however, this information was provided by only 64.7% of parents (n = 66). Most parents (92.2%, n = 94) reported that their child had not experienced severe hypoglycemic episodes, whereas 7.8% (n = 8) reported at least one such episode.

### 2.3. Instruments

#### 2.3.1. Sociodemographic Data

For this study a brief questionnaire was developed to collect demographic data from the participants, including age and sex of the parents and children; the parents’ and children’s educational level; their socioeconomic and marital status; the duration of the children’s T1D diagnosis, and any previous severe hypoglycemia events.

#### 2.3.2. Hypoglycemia Fear Survey—Parents (HFS—P)

The Hypoglycemia Fear Survey—Parents (HFS—P) is a modified version of the original Hypoglycemia Fear Survey developed by Clarke et al. (1998) (Portuguese version: Costa et al., 2025). The HFS—P consists of 26 items divided into two subscales: Behavior and Worry. The Behavior subscale assesses parental behaviors aimed at preventing hypoglycemic episodes that may be considered maladaptive, with items such as “*Giving my child a large snack at bedtime*” (Clarke et al., 1998). The Worry subscale includes the final 15 items and assesses parents’ fear and anxiety regarding the negative consequences of hypoglycemia and concerns about the possibility of hypoglycemic episodes in their children, with items such as “*My child having low blood sugar while sleeping*.”

Subscale scores are calculated by summing the corresponding items, and a total score is obtained by summing all items, with higher scores indicating greater FoH. In the original validation study, internal consistency was good for the Worry subscale (Cronbach’s α = 0.88) and moderate for the Behavior subscale (α = 0.72) (Clarke et al., 1998). Items are rated on a 5-point Likert scale ranging from 0 (never) to 4 (almost always). In the present study, the HFS—P showed excellent internal consistency for the total score (Cronbach’s α = 0.90; McDonald’s ω = 0.90). For the subscales, internal consistency was moderate for Behavior (α = 0.70; ω = 0.68) and excellent for Worry (α = 0.94; ω = 0.95).

#### 2.3.3. The Problem Areas in Diabetes Survey—Parent Revised Version (PAID—PR)

The Problem Areas in Diabetes—Parent Revised (PAID—PR) is an adaptation developed by Markowitz et al. (2012) to assess diabetes-related distress in parents of children with T1D, specifically capturing perceived burden and emotional distress associated with the demands of daily diabetes management (Polonsky et al., 1995). The questionnaire comprises 18 items organized into two factors, each with nine items. Factor 1, Immediate Burden, reflects daily and short-term concerns related to caregiving demands and intensive disease management, with items such as “*I feel worn out by the constant effort to manage diabetes*.” Factor 2, Theoretical Burden, captures worries, negative emotions, and anxiety about the child’s future, including feelings of helplessness, guilt, fear, and emotional suffering related to long-term consequences, with items such as “*I worry about the future and the possibility that my child will have serious complications*.”

Total PAID—PR scores are obtained by summing all 18 items (score ranging from 5 to 90), and factor scores are calculated by summing the corresponding items, with higher scores indicating greater diabetes-related parental burden and emotional distress. Items are rated on a Likert scale ranging from 1 (not at all distressing) to 5 (extremely distressing).

In the original validation study, the PAID—PR showed good internal consistency for the total score (Cronbach’s α = 0.87), moderate internal consistency for Immediate Burden (α = 0.78), and good internal consistency for Theoretical Burden (α = 0.83) (Markowitz et al., 2012). In the present study, it demonstrated good internal consistency for the total score (α = 0.86; McDonald’s ω = 0.85).

#### 2.3.4. Emotion Regulation Questionnaire (ERQ)

The Emotion Regulation Questionnaire (ERQ) was developed by Gross and John (2003) (Portuguese version: Machado Vaz et al., 2008) to assess two emotion regulation (ER) strategies: Cognitive Reappraisal (CR) and Expressive Suppression (ES). The instrument comprises two subscales corresponding to these strategies. The CR subscale includes six items assessing individuals’ ability to change how they think about situations that may elicit negative emotions, thereby reducing their emotional impact, with items such as “*When I want to feel less negative emotion, I change the way I am thinking about the situation*.” The ES subscale consists of four items assessing the tendency to inhibit emotional expression or minimize outward emotional displays, with items such as “*I control my emotions by not expressing them*.”

Items are rated on a 7-point Likert scale ranging from 1 (strongly disagree) to 7 (strongly agree) (Gross & John, 2003). Subscale scores are calculated by summing the corresponding items, with higher scores indicating more frequent use of the respective strategy. In the present study, internal consistency was good for CR (α = 0.82; McDonald’s ω = 0.82) and moderate for ES (α = 0.75; ω = 0.77).

### 2.4. Procedures

All study procedures were approved by the Ethics Committee of Ispa-Instituto Universitário (Approval No. D-080-4-24). Before completing the questionnaires, participants were informed about the aims and purpose of the study and provided informed consent, which guaranteed voluntary participation, anonymity, and the right to withdraw at any time during participation without consequences. No financial or other incentives were offered for participation.

### 2.5. Data Analysis

Data analyses were conducted using IBM SPSS Statistics (version 30, Chicago, IL, USA) and the PROCESS macro for SPSS (version 4.3, Calgary, AB, Canada) (Hayes, 2015). Descriptive statistics were first computed for sociodemographic variables to characterize the sample and to examine distributional assumptions, followed by descriptive statistics for all study measures. Acceptable values for assuming univariate normality were defined as skewness ≤ 2 and kurtosis ≤ 7 (Curran et al., 1996; West et al., 1995).

Internal consistency of the instruments was assessed using Cronbach’s alpha (α) and McDonald’s omega (ω). Alpha values were interpreted as follows: ≥0.90, excellent; ≥0.80, good; ≥0.70, acceptable; ≥0.60, questionable; and <0.60, poor (Aspinwall et al., 2016). Pearson correlation coefficients were calculated to examine associations between variables, and effect size magnitude was interpreted as small (|r| = 0.10–0.29), moderate (|r| = 0.30–0.49), and large (|r| ≥ 0.50), following Dancey and Reidy (2017).

Exploratory mediation analyses were conducted using PROCESS (Model 4), with Cognitive Reappraisal (CR) and Expressive Suppression (ES) as independent variables (tested in separate models), the Behavior and Worry dimensions of fear of hypoglycemia as mediators representing parental mental health, and Diabetes DD as the dependent variable. Total, direct, and indirect effects were estimated using unstandardized regression coefficients. The significance of indirect effects was evaluated using bias-corrected bootstrap confidence intervals (95%) based on 5000 resamples, with effects considered statistically significant when the confidence interval did not include zero. For all other analyses, statistical significance was set at *p* < 0.05.

## 3. Results

### 3.1. Descriptive Statistics

Descriptive statistics are presented in Table 1. The instrument assessing DD (PAID—PR) showed a maximum observed score of 89 (of 90), with a mean of 56.21 (SD = 17.65). The total score of the Hypoglycemia Fear Survey—Parents (HFS—P) reached a maximum of 88, with a mean of 45.70 (SD = 15.84). For the Behavior subscale (B-HFS—P), the mean was 24.64 (SD = 5.66), with a maximum score of 40, whereas for the Worry subscale (W-HFS—P), the mean was 21.06 (SD = 13.34), with a maximum score of 54. For ER strategies, mean scores were 3.62 (SD = 1.36) for Expressive Suppression and 4.74 (SD = 1.10) for Cognitive Reappraisal, both on a scale ranging from 1 to 7.

Skewness and kurtosis values for all variables were within acceptable limits, indicating no substantial departures from normality (Table 1).

### 3.2. Correlations

Correlations among study variables are presented in Table 2. Diabetes Distress was positively and significantly associated with total Fear of Hypoglycemia, as well as with both the Behavior and Worry subscales, with small effect sizes, indicating that higher levels of hypoglycemia-related fear were related to greater diabetes-related distress.

Expressive Suppression was not significantly associated with Diabetes Distress, total Fear of Hypoglycemia, or either of its subscales. In contrast, Cognitive Reappraisal was negatively and significantly associated with total Fear of Hypoglycemia and with the Worry subscale, suggesting that greater use of cognitive reappraisal was related to lower levels of parental worry about hypoglycemia. However, Cognitive Reappraisal was not significantly associated with Diabetes Distress or with the Behavior subscale of Fear of Hypoglycemia. No significant association was observed between Cognitive Reappraisal and Expressive Suppression.

### 3.3. Indirect Effects

#### 3.3.1. Model 1: Mediation Analysis of Behavior in the Association Between Cognitive Reappraisal and Diabetes Distress

The direct pathway of CR on DD was negative but not statistically significant, path c’ (B = −0.13, SE = 0.27, *p* = 0.635, 95% CI [−0.65, 0.40]). Although Behavior showed a positive and statistically significant association with DD, path b (B = 0.84, SE = 0.31, *p* < 0.01, 95% CI [0.23, 1.44]), CR was not significantly associated with Behavior, path a (B = −0.15, SE = 0.09, *p* = 0.093, 95% CI [−0.32, 0.02]), accounting for only 2.8% of the variance in Behavior (R^2^ = 0.03, F(1, 100) = 2.87, *p* = 0.093).

The full mediation model including both CR and Behavior explained 7.9% of the variance in DD (R^2^ = 0.08, F(2, 99) = 4.24, *p* < 0.05). The total pathway of CR on DD was also negative and non-significant, path c (B = −0.25, SE = 0.27, *p* = 0.360, 95% CI [−0.78, 0.29]), with CR explaining less than 1% of the variance in DD (R^2^ = 0.01, F(1, 100) = 0.85, *p* = 0.360).

**Figure 1:**
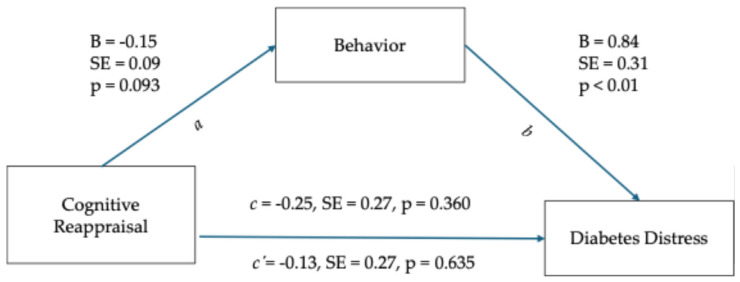
Mediation analysis of Behavior in the association between Cognitive Reappraisal and Diabetes Distress.

Bootstrap analyses indicated that the indirect pathway of CR on DD via Behavior was not statistically significant, as the 95% bias-corrected confidence interval included zero (indirect effect = −0.12, 95% CI [−0.32, 0.02]). Therefore, there was no evidence that Behavior mediated the association between Cognitive Reappraisal and Diabetes Distress (see Figure 1).

#### 3.3.2. Mediation Analysis of Worry in the Association Between Cognitive Reappraisal and Diabetes Distress

**Figure 2:**
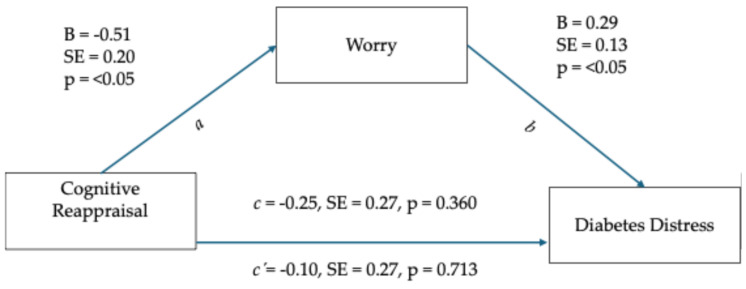
Mediation analysis of Worry in the association between Cognitive Reappraisal and Diabetes Distress.

Figure 2 presents the mediation model examining the role of the Worry subscale of Fear of Hypoglycemia in the association between Cognitive Reappraisal (CR) and Diabetes Distress (DD). The direct pathway of CR on DD was negative but not statistically significant, path c’ (B = −0.10, SE = 0.27, *p* = 0.713, 95% CI [−0.64, 0.44]). Worry showed a positive and statistically significant association with DD, path b (B = 0.29, SE = 0.13, *p* = <0.05, 95% CI [0.03, 0.56]).

The pathway of CR on Worry was negative and statistically significant, path a (B = −0.51, SE = 0.20, 95% CI [−0.90, −0.11]), accounting for 6.1% of the variance in Worry (R^2^ = 0.06, F(1, 100) = 6.53, *p* < 0.05). However, the overall mediation model including both CR and Worry was not significantly associated with DD, explaining 5.4% of the variance (R^2^ = 0.05, F(2, 99) = 2.81, *p* = 0.065). The total effect was non-significant (B = −0.25, SE = 0.27, *p* = 0.360, 95% CI [−0.78, 0.29]).

Despite the non-significant overall model, bootstrap analyses indicated that the indirect pathway of CR on DD through Worry was negative and statistically significant (indirect effect = −0.15, 95% CI [−0.34, −0.02]). This finding suggests that Worry partially explains the association between Cognitive Reappraisal and Diabetes Distress.

#### 3.3.3. Mediation Analysis of Behavior in the Association Between Expressive Suppression and Diabetes Distress

**Figure 3:**
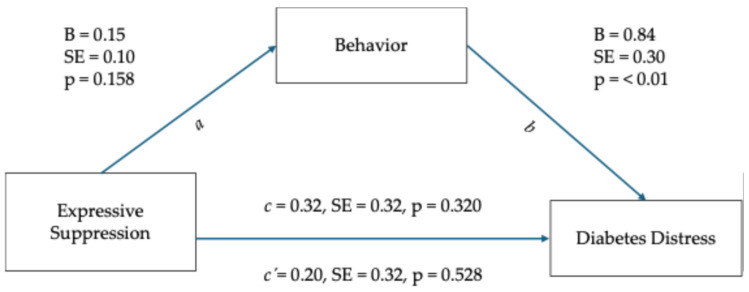
Mediation analysis of Behavior in the association between Expressive Suppression and Diabetes Distress.

Figure 3 presents the mediation model examining the role of the Behavior subscale of Fear of Hypoglycemia in the association between Expressive Suppression (ES) and Diabetes Distress (DD). The direct pathway of ES on DD was positive but not statistically significant, path c’ (B = 0.20, SE = 0.32, *p* = 0.528, 95% CI [−0.43, 0.83]). Although Behavior showed a positive and statistically significant association with DD, path b (B = 0.84, SE = 0.30, *p* = < 0.01, 95% CI [0.23, 1.4]). The pathway of ES on Behavior was positive but not statistically significant, path a (B = 0.15, SE = 0.10, *p* = 0.158, 95% CI [−0.06, 0.35]), accounting for only 2% of the variance in Behavior (R^2^ = 0.02, F(1, 100) = 2.02, *p* = 0.158).

The mediation model including both ES and Behavior explained 8% of the variance in DD (R^2^ = 0.08, F(2, 99) = 4.33, *p* < 0.05). The total pathway of ES on DD was also not statistically significant, path c (B = 0.32, SE = 0.32, *p* = 0.320, 95% CI [−0.32, 0.97]), with ES explaining approximately 1% of the variance in DD (R^2^ = 0.01, F(1, 100) = 1.00, *p* = 0.320).

Bootstrap analyses indicated that the indirect pathway of ES on DD via Behavior was not statistically significant, as the 95% confidence interval included zero (indirect effect = 0.12, 95% CI [−0.07, 0.36]). Therefore, there was no evidence that Behavior mediated the association between Expressive Suppression and Diabetes Distress.

#### 3.3.4. Mediation Analysis of Worry in the Association Between Expressive Suppression and Diabetes Distress

**Figure 4:**
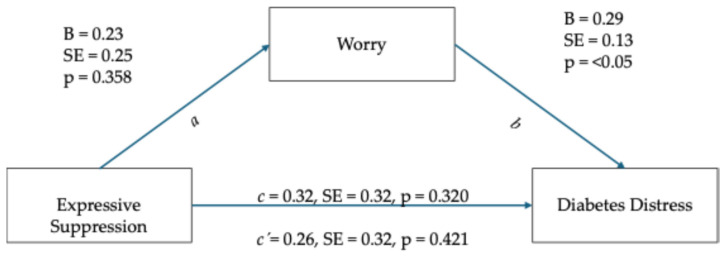
Mediation analysis of Worry in the association between Expressive Suppression and Diabetes Distress.

Figure 4 presents the mediation model examining the role of the Worry subscale of Fear of Hypoglycemia in the association between Expressive Suppression (ES) and Diabetes Distress (DD). The direct pathway of ES on DD was positive but not statistically significant, path c’ (B = 0.26, SE = 0.32, *p* = 0.421, 95% CI [−0.38, 0.89]). Worry showed a positive and statistically significant association with DD, path b (B = 0.29, SE = 0.13, *p* = <0.05, 95% CI [0.04, 0.55]).

The pathway of ES on Worry was positive but not statistically significant, path a (B = 0.23, SE = 0.25, *p* = 0.358, 95% CI [−0.26, 0.71]), explaining less than 1% of the variance in Worry (R^2^ = 0.01, F(1, 100) = 0.85, *p* = 0.358). The overall mediation model including both ES and Worry did not significantly explain DD, accounting for approximately 6% of the variance (R^2^ = 0.06, F(2, 99) = 3.08, *p* = 0.051). The total effect was not significant (B = 0.32, SE = 0.32, *p* = 0.320, 95% CI [−0.32, 0.96]).

Bootstrap analyses indicated that the indirect pathway of ES on DD through Worry was not statistically significant (indirect effect = 0.07, 95% CI [−0.08, 0.23]). Therefore, there was no evidence that Worry mediated the association between Expressive Suppression and Diabetes Distress.

## 4. Discussion

The increasing incidence of T1D in children and adolescents worldwide—in Europe (International Diabetes Federation, 2025), and in Portugal (Do Vale et al., 2024)—highlights the need and the importance of understanding how Fear of Hypoglycemia (FoH) relates to Emotion Regulation (ER) strategies and Diabetes Distress (DD) in parents, who are the primary caregivers, and to develop psychological interventions tailored to this population. Despite its clinical relevance, few studies have examined these variables jointly in parents of children with T1D. Accordingly, the present exploratory study aimed to examine how FoH (Behavior and Worry dimensions) is associated with the relationship between ER strategies, namely Cognitive Reappraisal (CR) and Expressive Suppression (ES), and DD in a relational framework, rather than assuming directional effects.

The hypothesis (H1), that greater use of CR would be associated with lower levels of DD, was not supported, indicating that more frequent use of CR was not directly associated with lower DD among parents, i.e., CR and DD do not show a direct relational pattern in this sample. Rather than suggesting a unidirectional pathway, this finding seems to reflect the absence of a straightforward association between general cognitive reappraisal tendencies and the multidimensional experience of DD among parents of children with T1D, explaining the nature of DD. DD encompasses not only emotional burden but also highly contextual and practical challenges related to daily disease management, including persistent concerns about future complications, perceived lack of support from health professionals, limitations in social support, and doubts about parental competence in managing the child’s condition (Dennick et al., 2017; Kreider, 2017; Streisand et al., 2001; Polonsky et al., 1995). Given this strong situational component, DD may show weaker relational alignment with cognitive strategies focused on internal emotional reinterpretation, such as CR (Gross, 2001).

In addition, CR is a cognitively demanding strategy that requires deliberate effort and regulatory resources (W. X. Toh et al., 2024). Within the context of chronic stress and intensive caregiving demands, different emotion regulation strategies may co-occur and be flexibly used depending on situational constraints and available cognitive resources (Sheppes & Meiran, 2008; Sheppes et al., 2014). Parents may therefore rely on a broader repertoire of emotion regulation and coping strategies, including those that are less cognitively demanding or more immediately accessible in daily routines, such as distraction, rumination and expressive suppression (Mor Keleynikov et al., 2023; Noser et al., 2019). This broader regulatory context may help explain the lack of a consistent relational association between CR and DD.

A more specific relational pattern emerged for the Worry dimension of FoH. According to the mediation analyses, H3 was partially supported through the Worry dimension. Greater use of CR was associated with lower levels of hypoglycemia-related worry, which was in turn associated with lower levels of DD. Importantly, this pattern should be interpreted as a co-occurrence of psychological constructs rather than a causal pathway. It highlights the central role of worry in the broader network of associations linking ER strategies and diabetes-related distress. This finding is consistent with previous research suggesting that parents who report greater use of adaptive ER strategies, such as CR, tend to report higher psychological resources, including lower exhaustion, higher confidence, and greater perceived efficacy in diabetes management, which may contribute to lower DD (Bassi et al., 2023; Coccaro et al., 2021; Kimbell et al., 2021). In this sense, the present results suggest that CR may be particularly relevant when parents experience elevated levels of hypoglycemia-related worry, as the use of cognitive reappraisal appears to reduce distress primarily by attenuating specific fears related to hypoglycemia rather than by directly reducing global diabetes-related burden.

Sample characteristics may also be relevant for understanding these relational patterns. A substantial proportion of parents had higher educational attainment (40.2% with an undergraduate degree) and most reported middle socioeconomic status (82.4%), factors that have been associated with greater access to cognitive and psychosocial resources supporting cognitively demanding regulation strategies (Burin et al., 2023; Z. Q. Toh et al., 2021). The absence of a direct pathway of CR on DD, together with the presence of a significant indirect pathway through Worry, reinforces the relevance of hypoglycemia-related worry as a proximal mechanism linking ER to diabetes distress. These contextual characteristics may shape how ER strategies and diabetes-related concerns co-occur in daily caregiving experiences, without implying directional pathways between constructs.

The Behavioral subscale did not show a mediating role in the association between CR and DD, contrary to H3. From a relational perspective, previous studies suggest that adaptive ER strategies such as CR may reduce FoH in parents (Kraaij & Garnefski, 2015; Zhang et al., 2022). This finding can be explained by the absence of a significant association between CR and hypoglycemia-related behaviors, indicating that greater use of CR was not associated with either increased or decreased engagement in avoidance behaviors aimed at preventing hypoglycemia. Nevertheless, Behavior was positively associated with DD, suggesting that these constructs tend to co-occur. Rather than reflecting a mechanistic pathway, this pattern may indicate that greater engagement in hypoglycemia-avoidance behaviors is part of a broader caregiving context characterized by increased monitoring demands and sustained responsibility for disease management, most of them recommended by healthcare professionals, which are themselves associated with higher DD (Abitbol & Palmert, 2021; Jensen et al., 2022). The lack of mediation by Behavior may therefore reflect the fact that CR primarily targets internal emotional and cognitive processes (Gross, 2001), rather than practical or behavioral aspects of disease management, such as preventive or avoidance behaviors related to hypoglycemia.

Furthermore, hypoglycemia-related behaviors are likely shaped by multiple factors beyond emotion regulation strategies, including medical recommendations and established diabetes-management routines (Abitbol & Palmert, 2021; Jensen et al., 2022). In this sense, such behaviors may reflect adaptive vigilance within clinical care rather than emotionally driven regulation processes. This dual function may explain their positive association with DD while not showing a systematic relational link with CR.

Another contextual factor is the clinical profile of the sample, where most parents (92.2%) reported that their child had not experienced severe hypoglycemic episodes. This may indicate that preventive behaviors are routinely implemented as part of standard care. Under these conditions, behavioral responses may be more closely aligned with medical guidance and habitual management practices than with emotion regulation tendencies, or emotional coping processes, reducing the likelihood of a systematic association between ER strategies such as CR and Behavioral avoidance of hypoglycemia.

Results from the third and fourth mediation models indicated that neither the Behavior nor the Worry dimensions of FoH were significantly associated with the relationship between ES and DD (H4) and no direct association was found between ES and DD (H2). Therefore, both hypotheses were not supported, meaning that more frequent use of ES would be associated with higher levels FoH and greater DD. From a relational perspective, these findings suggest that ES, FoH, and DD did not show consistent patterns of co-occurrence in this sample. Rather than indicating the absence of relevance of ES, the findings point to a weak or non-systematic associations between expressive suppression and diabetes-related emotional experiences among parents of children with T1D or the parental inhibition of emotional expression does not increase DD through either greater engagement in hypoglycemia-avoidance behaviors or higher levels of hypoglycemia-related worry.

The absence of significant pathways of ES was more likely related to the functional characteristics of ES itself, along with the specific characteristics of the ER questionnaire and the nature of DD and FoH. ES primarily targets the behavioral expression of emotions and operates at a later stage of the emotion-generative process, after emotional responses have already been activated (Gross, 2001; Gross & John, 2003; John & Gross, 2004). In contrast, worry about hypoglycemia reflects an anticipatory cognitive–emotional process related to perceived threat and uncertainty regarding future episodes. Therefore, it is theoretically consistent that ES would not be strongly associated with hypoglycemia-related worry, as suppression does not directly influence threat appraisal or anticipatory concerns.

Similarly, the absence of mediation through Behavior suggests that hypoglycemia-avoidance behaviors are not driven by emotional inhibition, but rather by medical recommendations, habitual management routines, or situational vigilance. This supports the interpretation that such behaviors may increase independently of the use of ES, which focuses on regulating emotional expression rather than guiding health-related behavioral decisions.

Another possible interpretation is that other psychological variables not assessed in the present study may show stronger associations with both FoH and DD than ES. For example, parental anxiety, which is highly prevalent among caregivers of children with T1D (Khemakhem et al., 2020), may be more closely associated with hypoglycemia-related worry, behavioral vigilance, and diabetes-related distress. Thus, the observed relational patterns may reflect the greater salience of diabetes-specific fears and caregiving demands relative to general ER tendencies such as expressive suppression.

The specific context of T1D may also be relevant for understanding these findings. The Emotion Regulation Questionnaire assesses general ER tendencies and is not specifically tailored to diabetes-related situations. Consequently, ES as measured in this study may not fully capture the emotional processes most closely linked to diabetes management experiences. In contrast, FoH, particularly its Worry and Behavior dimensions, appeared to show stronger and more consistent associations with DD across the models, being the primary contributor to DD. This pattern is consistent with previous studies demonstrating that parents of children and adolescents with T1D frequently report high levels of FoH, which is associated with greater DD, poorer psychological well-being and lower quality of life (Abitbol & Palmert, 2021; Driscoll et al., 2016; Patton et al., 2011, 2019).

Overall, DD in this sample appeared to be more strongly associated with the continuous practical demands of daily diabetes management than with parents’ internal ER processes, particularly expressive suppression. Persistent concerns regarding glycemic control, prevention of hypoglycemia, and ongoing monitoring requirements may constitute central aspects of the caregiving experience that co-occur with increased DD (Rodríguez-Muñoz et al., 2024). Within this relational framework, FoH emerges as a more proximal correlate of DD than ES.

Finally, the gender composition of the sample should be considered as one contextual characteristic that may have shaped the observed relational patterns. The sample consisted predominantly of mothers (92.2%). Previous research indicates that men tend to use expressive suppression more frequently than women (Gross & John, 2003), which may partly explain why ES was not significantly associated with either FoH or DD in this study. Furthermore, mothers have been shown to report higher levels of both behavioral and worry-related components of FoH compared to fathers (Allen et al., 2024; Haugstvedt et al., 2010; Pate et al., 2016), which may have strengthened the observed associations between FoH and DD in models including ER strategies. 

## 5. Limitations

Despite its contributions, this study has some limitations that should be considered when interpreting the findings. First, the sample size was relatively small (N = 102), which may have limited statistical power, particularly for detecting small indirect effects in mediation analyses. In addition, the sample was predominantly composed of mothers with relatively high educational attainment and mostly middle socioeconomic status, which restricts the generalizability of the results to more socioeconomically diverse families and to fathers, who may experience and manage diabetes-related stress differently.

Second, participants were recruited through social media groups using convenience sampling, which may have introduced self-selection bias. Parents who are more engaged in online support communities or more concerned about their child’s condition may have been more likely to participate, potentially inflating estimates of FoH or DD. Third, all data were collected using self-report measures, which are subject to response biases, including social desirability and recall bias. Moreover, the use of a single informant per family prevents examination of discrepancies between caregivers or between parental reports and clinical indicators of disease management. The online format of data collection also limited control over the conditions under which questionnaires were completed and precluded verification of clinical information such as glycemic control or medical history.

Fourth, the cross-sectional design precludes any conclusions about causal directionality. Although mediation models were tested, the temporal ordering of ER strategies, FoH, and DD cannot be established. Longitudinal designs are needed to determine whether changes in ER precede changes in fear and distress, or whether elevated distress influences the use of specific ER strategies over time. Fifth, the low proportion of parents reporting severe hypoglycemic episodes (7.8%) may have reduced variability in fear-related responses, particularly in the Behavior subscale, potentially limiting the detection of stronger associations between fear of hypoglycemia, emotion regulation, and diabetes distress.

Finally, the Emotion Regulation Questionnaire assesses general regulation strategies and is not specific to health-related or diabetes-related emotional contexts. This may have limited its sensitivity to capture ER processes that are more directly linked to disease-specific stressors. Future studies could benefit from using or developing diabetes-specific ER measures or combining self-report with behavioral or ecological momentary assessment methods to better capture real-time regulation processes in daily caregiving situations.

## 6. Practical Implications

One of the main contributions of this study lies in providing updated and context-specific information, that may help inform psychological support approaches for parents of children and adolescents with T1D. Although the observed effect sizes and explained variance were relatively small, the findings suggest that FoH, particularly hypoglycemia-related worry, may represent a relevant factor associated with DD. Previous research has evidenced that a substantial percentage of DD appears to be related to FoH (Li et al., 2021) and this fear is associated with anxiety, depressive symptoms, and DD (Zhang et al., 2022), as well as with parental emotion regulation skills and positive self-appraisal (Aalders et al., 2018).

In the present study, hypoglycemia-related worry emerged as a potential mechanism linking CR to DD, suggesting that interventions addressing anticipatory fears related to hypoglycemic episodes may be beneficial for parents. This is consistent with previous findings indicating that parental worry about hypoglycemia is associated with higher DD and poorer quality of life (Abitbol & Palmert, 2021; Patton et al., 2007). Therefore, psychotherapeutic interventions targeting FoH and related anxiety symptoms may contribute to reducing diabetes-related distress (Patton et al., 2011). Nevertheless, these findings should be interpreted cautiously as the magnitude of the observed associations is modest.

Cognitive–behavioral therapy (CBT) approaches appear particularly suitable, as they have demonstrated effectiveness in enhancing ER skills and in reducing anxiety and anger associated with DD and FoH in caregivers (Silva, 2023). CBT-based interventions could incorporate components such as cognitive restructuring of catastrophic beliefs about hypoglycemia, exposure to uncertainty, and skills training for managing anxiety during high-risk situations (e.g., nighttime monitoring). Integrating psychoeducation with ER training may also strengthen parents’ sense of competence and reduce maladaptive vigilance behaviors.

## 7. Conclusions

The present study aimed to examine whether FoH explains the associations between ER strategies, namely CR and ES and DD, through its Behavioral and Worry dimensions. Overall, the findings provide partial support for this integrative model and highlight FoH, particularly hypoglycemia-related worry, as a key mechanism underlying parental diabetes distress. This is an exploratory study since ER is not usually explored in the context of T1D and, for that reason, results should be interpreted with caution.

Contrary to expectations, CR was not directly associated with lower DD. However, a significant indirect effect emerged through the Worry dimension of FoH, indicating that greater use of CR was associated directly with reduced hypoglycemia-related worry, which in turn was linked to lower DD. This pattern suggests that CR may contribute to reduced distress primarily by attenuating specific fear-based cognitions rather than by influencing overall diabetes-related burden. In contrast, the Behavioral dimension of FoH did not mediate this association, indicating that CR is more strongly related to internal cognitive-emotional processes than to diabetes-related preventive behaviors.

Regarding ES, results did not support either direct or indirect associations with DD. Neither Worry nor Behavior dimensions mediated the relationship between ES and DD, suggesting that ES does not meaningfully contribute to DD in this context. This may reflect the fact that ES primarily influences outward emotional expression than other psychological processes which perhaps can be more directly relevant to FoH and DD.

Importantly, FoH, particularly its Worry dimension, emerged as the most consistent contributor to DD across models, reinforcing its central role in the emotional experience of parents managing pediatric T1D. Overall, the findings suggest that DD is more closely linked to diabetes-specific fears and daily management demands, than to general emotion regulation tendencies. These results underscore the importance of targeting hypoglycemia-related worry in psychological interventions aimed at reducing DD in T1D care.

## Figures and Tables

**Table 1:** Descriptive statistics of the study variables (N = 102).

	M	SD	Minimum	Maximum	Skewness	Kurtosis
Diabetes Distress	56.21	17.65	5	89	−0.416	−0.325
FoH—Total score	45.70	15.84	20	88	0.728	−0.086
FoH—Behavior	24.64	5.66	13	40	0.065	−0.466
FoH—Worry	21.06	13.34	5	54	0.098	−0.238
Expressive suppression	3.62	1.36	1	7	−0.034	−0.696
Cognitive reappraisal	4.74	1.10	2	7	0.123	−0.256

Note: SD = Standard Deviation; M = Mean.

**Table 2:** Pearson correlations between the study variables.

Variables	1	2	3	4	5
1. Diabetes distress	-	-	-	-	-
2. FoH—Total score	0.292 **	-	-	-	-
3. FoH—Behavior	0.277 **	0.585 ***	-	-	-
4. FoH—Worry	0.229 *	0.939 ***	0.271 **	-	-
5. Expressive suppression	0.099	0.128	0.141	0.092	-
6. Cognitive reappraisal	−0.092	−0.268 **	−0.167	−0.248 *	0.028

Note. * *p* < 0.05; ** *p* < 0.01; *** *p* < 0.001.

## Data Availability

Data is unavailable due to privacy or ethical restrictions.

## Abbreviations

The following abbreviations are used in this manuscript:

T1D	Type 1 Diabetes
SPSS	Statistical Package for Social Sciences
DD	Parental Diabetes Distress
ER	Emotional Regulation
CR	Cognitive Reappraisal
ES	Expressive Suppression
FH	Fear of Hypoglycemia
HFSP—P	Hypoglycemia Fear Survey—Parents
PAID—PR	The Problem Areas in Diabetes Survey—Parent Revised Version
ERQ	Emotional Regulation Questionnaire
α	Alpha de Cronbach
ϖ	Ômega de McDonald
